# Machine Learning and Pharmacogenomics at the Time of Precision Psychiatry

**DOI:** 10.2174/1570159X21666230808170123

**Published:** 2023-09-25

**Authors:** Antonio Del Casale, Giuseppe Sarli, Paride Bargagna, Lorenzo Polidori, Alessandro Alcibiade, Teodolinda Zoppi, Marina Borro, Giovanna Gentile, Clarissa Zocchi, Stefano Ferracuti, Robert Preissner, Maurizio Simmaco, Maurizio Pompili

**Affiliations:** 1 Department of Dynamic and Clinical Psychology and Health Studies, Faculty of Medicine and Psychology, Sapienza University; Unit of Psychiatry, ‘Sant’Andrea’ University Hospital, Rome, Italy;; 2 Department of Neuroscience, Mental Health and Sensory Organs (NESMOS), Faculty of Medicine and Psychology, Sapienza University; Unit of Psychiatry, ‘Sant’Andrea’ University Hospital, Rome, Italy;; 3 Department of Neuroscience, Mental Health and Sensory Organs (NESMOS), Faculty of Medicine and Psychology, Sapienza University; Unit of Laboratory and Advanced Molecular Diagnostics, ‘Sant’Andrea’ University Hospital, Rome, Italy;; 4 Department of Human Neuroscience, Faculty of Medicine and Dentistry, Sapienza University, Unit of Risk Management, ‘Sant’Andrea’ University Hospital, Rome, Italy;; 5 Institute of Physiology and Science-IT, Charité - Universitätsmedizin Berlin, Corporate Member of Freie Universität Berlin, Humboldt-Universität zu Berlin, and Berlin Institute of Health, Philippstrasse 12, 10115, Berlin, Germany

**Keywords:** Machine learning, pharmacogenomics, artificial intelligence, precision psychiatry, precision medicine, traditional medicine

## Abstract

Traditional medicine and biomedical sciences are reaching a turning point because of the constantly growing impact and volume of Big Data. Machine Learning (ML) techniques and related algorithms play a central role as diagnostic, prognostic, and decision-making tools in this field. Another promising area becoming part of everyday clinical practice is personalized therapy and pharmacogenomics. Applying ML to pharmacogenomics opens new frontiers to tailored therapeutical strategies to help clinicians choose drugs with the best response and fewer side effects, operating with genetic information and combining it with the clinical profile. This systematic review aims to draw up the state-of-the-art ML applied to pharmacogenomics in psychiatry. Our research yielded fourteen papers; most were published in the last three years. The sample comprises 9,180 patients diagnosed with mood disorders, psychoses, or autism spectrum disorders. Prediction of drug response and prediction of side effects are the most frequently considered domains with the supervised ML technique, which first requires training and then testing. The random forest is the most used algorithm; it comprises several decision trees, reduces the training set's overfitting, and makes precise predictions. ML proved effective and reliable, especially when genetic and biodemographic information were integrated into the algorithm. Even though ML and pharmacogenomics are not part of everyday clinical practice yet, they will gain a unique role in the next future in improving personalized treatments in psychiatry.

## 1 INTRODUCTION

The biomedical sciences have always been profoundly and strongly dependent on data, even more in the latest decades with precision medicine's birth and development, which caused the need to collect an increasing number of complex and multi-dimensional data. All this amount of data derives from both microscopic and macroscopic worlds, which led to the need to process a massive amount of information emanating from very different biological contexts and systems [1, 2].

Another distinction lies in the difference between biological data and clinical variables, such as patients' records, lifestyles, and psychological aspects. Contemporary medical sciences require new and practical tools to gather and process all the acquired and produced clinical data to give the most detailed representation of complex pathophysiological processes [3]. Therefore, scientists must face dimensional heterogeneities and categorical distinctions that cannot be performed clinically and require analysis with complex software systems.

Today we are witnessing the Big Data era in its early stage of development since most of the technologies, practices, and analytical applications appeared around 2010 [4]. Big Data means a massive amount of digital data collected from any sources that are raw, unstructured, and too different from each other to be analyzed using conventional statistical and relational techniques [5]. All the features of Big Data can be summarized with “the three V's”: volume, velocity, and variety. First, volume refers to the massive quantity of data; each organization generates terabytes or petabytes of information. Second, variety describes the different natures of the data themselves. Third, velocity is linked with the insane frequency with which today's data is generated, gathered, and processed. All the value of these data loses importance without an effective system for managing, extracting, and analyzing it [5]. The systematic and comprehensive exploration of data is mainly carried out using Artificial Intelligence which provides a mechanism for data-driven hypotheses, experimental planning, precision, and evidence-based medicine.

### 1.1 Precision Psychiatry

Psychiatry is dedicated to understanding mental diseases and assisting those affected in leading gratifying lives. Although current treatment strategies for many mental disorders can be remarkably effective at improving patients' quality of life and mitigating the burden of symptoms, finding the proper treatment for an individual can be a long and arduous process, during which symptoms can worsen and could increase the clinical risk related to other health conditions.

Precision psychiatry is a promising new direction to overcome those limitations [6]. It consists of the translation into the clinical psychiatry of the precision medicine methods, thus considering the latest biomarker-based research approaches to accurately assess an individual's risk of developing mental illnesses for preventive purposes. Predictive psychiatry aims to construct clinical and molecular models to better predict individual and varied therapy responses and increase the early detection of mental diseases. Pattern recognition could extract signatures from clinical, cognitive, imaging-based, and, where applicable, genetic data that can be applied quantitatively to individual patients to anticipate desired and undesired pharmacological effects. Another main objective of precision psychiatry is to identify drug treatments that could have better efficacy and tolerability for a specific patient [7].

The most recent innovations come from the fields of pharmacogenomics and Artificial Intelligence, of which ML is currently among the most promising approaches.

### 1.2 Machine Learning

ML is made up of mathematics, statistic, and computer science. It can be considered an engine, a kind of “intelligent” product whose ability is to make accurate and precise predictions based on data from several different sources [8]. ML techniques use algorithms that describe the relationships between variables. These algorithms might be represented on a continuum between easy to decode and understand and those with great difficulties in decoding; the whole working system may be compared to a “black box” [9].

Conventional statistical techniques, such as linear and logistic regression, can show the relationship between two variables; then, the inference is about how two data are related. On the other side, ML's primary goal is prediction; here, the main purpose is to assess whether and to what extent some data might predict an event [10]. The learning process has a crucial role in achieving a predictive capability and divides ML into two categories: Supervised ML and Unsupervised ML.

Supervised ML is a technique in which a model is trained on a range of features associated with a known outcome. These features might be represented by patients' characteristics or history related to a specific outcome (*e.g*., weight, BMI, and the onset of diabetes within some years). Once an algorithm is trained, it will predict outcomes when applied to a new data set. Furthermore, predictions can be discrete (*e.g*., healthy/unhealthy, malignant/benign) or continuous (*e.g*., range of values) [11, 12]. Both features and outcomes are organized in a dataset to which an algorithm may be applied. Then the algorithm is improved during its development to be optimized, reducing the risk of giving errors in predictions.

Unsupervised ML, so far, has found few applications in medicine [13]. Focusing on unsupervised ML, the main difference with supervised learning is the absence of a predefined outcome. The algorithm gains an exploratory purpose in this situation since the user does not include any output in the dataset. Hence, this kind of learning may have significant implications regarding the most complex pathophysiologic mechanisms and possible new therapeutic paths; on the other hand, the learning process is more difficult to understand and apply in clinical practice. Therefore, due to the inherent unpredictability of the results provided, the application of unsupervised ML in clinical practice still has several issues.

### 1.3 Machine Learning in Pharmacogenomics

Pharmacogenomics is widely considered one of the most promising fields of clinical medicine [14]; it focuses on identifying genomic aspects that could be correlated with drug effects and metabolization. Pharmacogenomics focuses on the role of the genome in drug response. It analyzes how the genetic asset of an individual can affect the response to drugs, having a potentially positive impact on clinical practice, primarily in treatment-resistant mental disorders [15]. The most prescribed psychiatric drug in 2015 was sertraline, a member of the selective serotonin reuptake inhibitors (SSRIs) class adopted for depression, obsessive-compulsive disorder, panic disorder, post-traumatic stress disorder, and anxiety disorders. This drug class includes many other molecules, such as fluvoxamine, citalopram, escitalopram, fluoxetine, and paroxetine, most of which demonstrated efficacy in 65% of treated patients or less [16, 17]. A similar issue exists in treating resistant schizophrenia spectrum disorders, for which the atypical antipsychotic drugs may show low or insufficient efficacy and response rates [18, 19]. These elements underline the need for a new paradigm in treating psychiatric disorders, switching from the traditional evidence-based approach (based on data gathered in large populations of patients) to an individual-based and data-driven knowledge of clinical and biological data (phenotypical, genotypical, and molecular). Thus, precision medicine's fundamental consists of tailoring care and focusing on the unique characteristics of patients [20].

Artificial intelligence and ML aim to provide a data-driven algorithm that learns from past and present data to elaborate predictive outcomes for any unknown data or any unknown event in the future [21, 22].

Thanks to the recent advances in multi-omics, precision psychiatry is acquiring high growth potential to satisfy the requirements of new drugs and therapeutic interventions [23]. Multi-omics currently promises to improve human health and disease knowledge, and many researchers are working on methods to generate and analyze disease-related data. Multi-omics applications improved understanding of host-pathogen interactions, infectious diseases, chronic and complex non-communicable diseases, and personalized medicine. However, the challenges we face today in precision psychiatry are still mostly unmatched, considering mental disorders' multifactorial aetiology. The development of software tools based on artificial intelligence and ML frameworks could help to predict specific quantitative and categorical phenotypes in clinical settings by utilizing next-generation technology multi-omics and neuroimaging datasets [24]. This study aims to systematically review the current evidence on the use of ML and artificial intelligence in precision psychiatry, underlining the current possibilities and promises of this approach for patients with mental disorders.

## 2 METHODS

A systematic review of the literature was conducted to investigate the field of application of ML technology in the study of pharmacogenomics in psychiatry, evaluating types and prospects of application. The examined studies were identified through research in online databases (PubMed, Scopus, Web of Science, CINHAL, and PsycINFO) carried out using the following string: ((machine learning) OR (deep learning) OR (algorithm)) AND pharmacogen* AND (psychiatr* OR mental).

We included articles describing studies focused on the use of ML and artificial intelligence in the field of pharmacogenomics in psychiatry. We excluded articles unrelated to the central issue of this review.

**Fig. (1):**
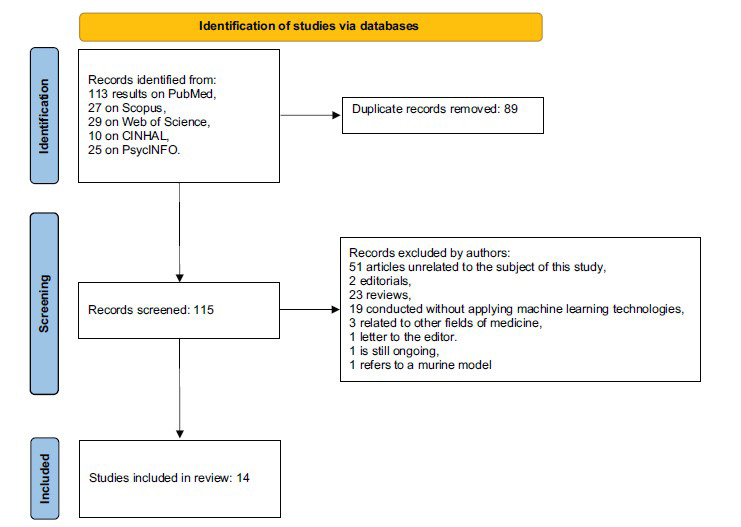
Search strategy.

The initial research was completed on November 28, 2021, producing 113 results on PubMed, 27 on Scopus, 29 on Web of Science, ten on CINHAL, 25 on PsycINFO. From these, 26 articles obtained from Scopus have been eliminated since they coincide with results obtained by PubMed; 28 from Web of Science, since similar articles in PubMed and Scopus; finally, ten articles from CINHAL and 25 from PsycINFO since already identified through the other search engines. Therefore, the preliminary investigation was conducted on 115 articles (113 from PubMed, 1 from Scopus, 1 from Web of Sciences). Among all, we excluded 51 articles unrelated to the object of this study, two editorials, 23 reviews, 19 that did not apply an ML method, three related to other fields of medicine, one letter to the editor, one animal study, and one ongoing study. Therefore, the total database included 14 peer-reviewed scientific articles (Fig. **1**).

## 3 RESULTS

### 3.1 Overview of Articles’ Characteristics

Our qualitative synthesis yielded 14 papers published between 2013 and 2021; this underlines the novelty of this topic and how the adoption of ML applied to pharmacogenomics is gaining interest in psychiatry. Out of 14, 11 studies have been published in the last three years [24-34]. The total sample is made up of 9, 180 patients with different diagnoses. 8 studies focused on Major depressive disorder (MDD) [24, 26, 27, 30, 32-35], 1 study on bipolar disorder (BD) [28], 1 study on MDD and depressive episodes in BD [31], three studies on psychotic spectrum diseases [25, 29, 36] and 1 on Autism spectrum disorder (ASD) [37]. Most of the papers (8) are cohort studies [24-26, 28-32], 2 of them are case-control [35, 36], and the other four are association studies derived from randomized control studies (RCT) [27, 33, 34, 37] (Tables **1** and **2**).

### 3.2 ML Application Domains in Pharmacogenomics

Two domains of pharmacogenomic ML in psychiatry were identified: (i) prediction of drug response (n=12) and (ii) prediction of side effects (n=2).

1) Prediction of drug response includes articles aiming to identify which genes and subpopulation characteristics are associated with a positive response to a specific drug class or molecule. According to recent studies, individual phenotypic differences may also emerge from epigenetic modifications like histone acetylation or DNA methylation. In addition, noncoding RNA interactions also have a role in protein expression and may alter drug effects. Different promising therapeutic techniques are now being developed, although the role of epigenetics in pharmacological treatment response needs further study [38]. The study of gene-gene interactions may better underline individual pharmacokinetic and pharmacodynamic pathways [21].

2) Prediction of side effects studies focuses on which genetic variables might play a role in the onset of undesirable effects due to a specific drug. In both domains, the core question is to find a suitable subpopulation, based on a genomic study, for a specific pharmacological treatment to establish a tailored therapy, theoretically, with no side effects and the best odds of a response.

### 3.3 Prediction of Drug Response

Eugene and colleagues focused on lithium treatment in bipolar and schizoaffective disorder; more specifically, they intended to spotlight the gender-specific transcriptional-level regulators of lithium treatment response [28]. They performed 4 Differential Gene Expression Analyses (DGEA). Through DGEA-1, the gender-specific transcriptome was obtained comparing male *vs.* female; DGEA-2 comparing male non-responders *vs.* male responders; DGEA-3 was performed on female non-responder *vs.* female responders and, finally, DGEA-4 on male responders *vs.* female responders. The main 250 genes from DGEA-1 to DGEA-4 were then overlaid to result in gender-linked genes related to the response to the treatment with lithium. After identifying the statistically significant DNA microarray genes, two ML algorithms were used for classification: Decision Tree and random forest. They selected the Decision Tree algorithm to classify male *versus* female samples; more specifically, the Ribosomal protein S4, Y-linked 1 (RPS4Y1) gene expression was ≥ 9.643 in male patients and < 9.643 in female patients with a probability=100%. A random forest algorithm was adopted for classifying male responders and female responders. The RBPMS2 and LILRA5 genes were involved in the lithium response in males with an area under the receiver operator characteristic curve (AUROC) of 0.92, and the ABRACL, FHL3, and NBPF14 genes were found related to female lithium responders with AUROC of 1. RBPMS2 is a gene codifying for an RNA Binding Protein with Multiple Splicing, while LILRA5 codifies for Leukocyte Immunoglobulin Like Receptor A5. ABRACL Codifies for ABRA C-Terminal-like protein, FHL3 for Four and a Half LIM Domains 3, and NBPF14 for Neuroblastoma Breakpoint Family Member 14.

ML-based algorithms analyzing functionally validated pharmacogenomic biomarkers associated with clinical measures could predict the remission/response rate to selective serotonin reuptake inhibitors (SSRIs) in patients affected by MDD [30]. Athreya *et al.* [30] studied 1, 030 MDD patients treated with citalopram/escitalopram from Mayo Clinic Pharmacogenomics Research Network Antidepressant Medication Pharmacogenomic Study (PGRN-AMPS; n = 398), Sequenced Treatment Alternatives to Relieve Depression (STAR*D; n = 467), and International SSRI Pharmacogenomics Consortium (ISPC; n = 165) trials. As pharmacogenomic biomarkers, they included six SNPs, either in or close to the TSPAN5 (rs10516436), ERICH3 (rs696692), DEFB1 (rs5743467, rs2741130, and rs2702877), and AHR (rs17137566) genes. SNPs were identified through a genome-wide association study for PGRN-AMPS plasma metabolites associated with SSRI response (serotonin) and baseline MDD severity (kynurenine) [30, 39, 40]. Unsupervised learning was applied to identify clusters of patients (men and women separately) with similar symptom severity at baseline and after 4 and 8 weeks of treatment. It was applied an Expectation-Maximization (EM) algorithm that assumed only one component in the mixture (a single bell-shaped curve distribution) and gradually increased the number of components (distributions with multiple peaks) until an adequate fit of the data was achieved. Then, they adopted a trained random forest algorithm (random forest R library) using PGRN-AMPS's baseline depression severity and pharmacogenomics data to predict remission/response and then externally validated by the trained prediction model using STAR*D and ISPC data. For both women and men, the top predictor for remission was baseline depression severity, followed by the DEFB1_2 (rs2741130) and DEFB1_1 (rs5743467) SNPs biomarkers identified during our GWAS for plasma kynurenine concentrations. The top SNPs for response in the men group were the TSPAN5 SNPs, related to serotonin concentration, followed by the DEFB1_1 and DEFB1_2 SNPs. For response in women, the top predictor was the DEFB1_1 SNP, followed by baseline depression severity and the DEFB1_2 SNP [30, 39, 40].

Another 6-week duration cohort study evaluated the therapeutic outcome of different antidepressants [32]. The expression of the C allele of rs6354 polymorphism and the G allele of rs12150214 (SLC6A4) showed a poorer treatment response to fluoxetine. The SNPs rs929377-rs6191-rs32897 were also significantly associated with the treatment response to fluoxetine. In female MDD patients, the minor allele of rs6323 and rs1137070 on the MAOA gene showed to be related to a worse response to venlafaxine.

Lin and colleagues tested a wrapper-based feature selection algorithm integrated with a boosting ensemble predictive framework for building predictive models of antidepressant treatment response among 421 MDD patients. Their primary purpose was to compare the efficacy of different ML techniques. This study demonstrated that the ensemble ML framework might be a valuable technique for creating bioinformatics tools for discriminating non-responders from responders before treatment with SSRIs [24].

Another study evaluated five different ML approaches (neural networks, recursive partitioning, learning vector quantization, Gradient boosted machine, and random forest) on three different samples testing 44 SNPs of 8 candidate genes (CACNA1C, CACNB2, ANK3, GRM7, TCF4, ITIH3, SYNE1, FKBP5). FKBP5 polymorphisms seemed effective candidates for inclusion in antidepressant pharmacogenetic tests. Furthermore, pathways including the CACNA1C, a Calcium channel-related gene, could be involved in treatment-resistant depression, which could be considered for developing multi-marker predictors [26].

Kautzky and colleagues focused their attention on treatment-resistant depression [35]. They demonstrated that using the random forest algorithm, combining SNPs (12 SNPs in HTR2A, COMT, ST8SIA2, PPP3CC, and BDNF) and clinical variables, it is possible to detect treatment-resistant patients. A combination of two ML models was tested by Maciukiewicz *et al.* [27], applying classification-regression trees (CRT) and linear support vector machine (SVM) to predict duloxetine response in MDD. Additionally, they used the genome-wide logistic regression to identify potentially significant SNPs variants related to duloxetine response/remission and extracted the most promising predictors using LASSO regression. CRT performed poorer for remission (accuracy = 0.51, sensitivity = 0.51, specificity = 0.51), when compared with SVM (accuracy = 0.52, sensitivity = 0.58, specificity = 0.46). In response, both algorithms performed poorly. Regarding CRT, models achieved an accuracy = 0.57, a sensitivity = 0.75, and a specificity = 0.15. In SVMs, they observed an accuracy = 0.64, a sensitivity = 0.87, and a specificity = 0.07. For remission, the SVM models achieved an accuracy = 0.41, a specificity = 0.43, and a sensitivity = 0.41. In conclusion, SVM models based on predefined classes perform significantly better.

Joyce and colleagues explored the application of ML tools in combined pharmacological treatment in MDD [34]. In detail, they examined data from 264 MDD patients treated with citalopram or escitalopram deriving from Mayo Clinic PGRN-AMPS and 111 MDD patients under treatment of a combined antidepressant therapy from Combined Medication to Enhance Outcomes of Antidepressant Therapy (CO-MED) study. The central hypothesis of Joyce and colleagues is that enriching clinical measures with biological ones (such as metabolomics and genomics) might improve the predictability of response to combined antidepressant therapies. They applied a first model made up of clinical, sociodemographic, and metabolomic (plasma metabolites) aspects and a second model, additionally considering six validated SNPs related to MDD pathophysiology and citalopram/escitalopram response. These SNP biomarkers are located near or in TSPAN5, ERICH3, DEFB1, and AHR genes [39-41]. Both linear and non-linear algorithms were tested. A linear regression model was successful at predicting changes in symptoms' scores using clinical and metabolomic features; they then tested extreme gradient-boosted decision tree-based ensembles (XGBoost) as nonparametric models. Nonparametric models identified possible non-linear relationships among predictors while predicting treatment outcomes. Finally, a cross-trial replication was conducted, showing that integrating data on specific metabolites and SNPs achieves more accurate treatment response predictions across classes of antidepressants [34].

Taliaz and his group used STAR*D patients' data for algorithm assembly and evaluation; they randomly divided 530 patients into a validation group of 271 and a test group of 259 [33]. They further proceeded with external validation of their ML tool, used on data from the PGRN-AMPS of patients treated with citalopram. They used several ML algorithms: SVM with a linear kernel, XGBoost, random forest, and Adaptive Boosting (AdaBoost). In addition, 5- or 10-fold repeated cross-validations (CVs) were performed on the training datasets to reach optimal parameters; these were used to re-train the various models using the complete training datasets. The authors considered 8, 210 SNPs, deriving from STAR*D genetic data and genetic data and the Genome Reference Consortium Human genome, obtaining highly similar results for STAR*D and PGRN-AMPS test sets, with good accuracy. These findings support the feasibility of using ML algorithms applied to large datasets with genetic, clinical, and demographic features to improve accuracy in antidepressant prescription [42].

The only study focused on the depressive symptoms among two different diagnosis groups (MDD and BD) was the one conducted by Borro *et al.* [31]. All the recruited patients were pharmacoresistant, with at least three previous failed treatments. A new algorithm-based tool, Drug-PIN, was employed to re-evaluate and optimize therapies. They compared results from Drug-PIN with the ones obtained by therapy counseling. The number of baseline poly-therapies classified as low-, moderate- or high-risk did not change significantly between the manual system or the Drug-PIN system. As the counseling process, also the Drug-PIN system showed a significant decrease in the predicted treatment-associated risk. In summary, this informatic tool seems to replicate traditional counseling, virtually reducing time and the risk of mistakes in everyday clinical practice.

Switching attention to antipsychotic drugs, Lee and colleagues developed a computational algorithm to personalize schizophrenia treatment [25]. This kind of algorithm was first adopted to identify who benefits most from the treatment group in clinical trials [43] and it is based on a classical clustering algorithm called the partitioning around medoids (PAM) algorithm. It uses both clinical profile and genetic information with two sets of SNPs. The authors proposed a computational algorithm that simultaneously used genetic information and clinical profiles to predict who will or will not benefit from a specific antipsychotic medication among patients with schizophrenia. The model provided a good prediction for Ziprasidone by 13 SNPs and 53 baseline variables [44, 45].

Only a study focused on ASD [37]. An SNP ranking algorithm was used based on a linear SVR with MATLAB 2014 on the LIBSVM package. The SNP information was binarized and divided into the SNP response data to test and train data in a leave one out cross-validation. The training data fitted the binary information of SNPs to the oxytocin efficacy with SVR and calculated the mean square error. This procedure was repeated for each SNP. The set of most informative SNPs based on the top 10% ranking was chosen. For SVR, there were 4 clusters, and calculated the weight of every SNP for each cluster. They evaluated the relationship between 27 OXTR SNPs and six types of behavioral/neural response to oxytocin treatment in 38 ASD patients. It came out that major alleles of several prominent OXTR SNPs, including the rs53576 and rs2254298, were related to the oxytocin effect. We resumed the main results of ML studies in psychiatry in Tables **1** and **2**.

### 3.4 Prediction of Side Effects

Boloc and colleagues used ML techniques to test whether it can predict the side effects of drug treatment, for example, the extrapyramidal symptoms that may occur during an antipsychotic treatment [29]. Supervision methods of class prediction based on ML were applied. ML has been trained to identify control and case classification patterns using the Discovery Sample of the SNPassoc R package. Support vector machine, Naive Bayes, and random forest were adopted, showing a better EPS prediction. The Naive Bayes achieved the best result. The exact purpose was pursued by Son *et al.* [36]. They investigated the polymorphisms associated with tardive dyskinesia in patients treated with typical antipsychotics and predicted it using the MDR (multifactor dimensionality reduction). MDR is nonparametric and model-free, made up of two stages. Stage 1 involves choosing the best combination of factors, and stage 2 involves classifying the combinations of genotypes into high-risk and low-risk groups based on the ratio of cases to controls with that genotype [46]. MDR ultimately selects one genetic model, single or multi-loci, which predicts phenotype with good success. The model's predictive ability was assessed using the 10-fold cross-validation. Statistical significance is determined empirically by permuting the case and control labels 1000 times. SCL6A11 genotypes distribution showed a significant difference between patients with and without tardive dyskinesia (TD), providing significant evidence for gene-gene interactions (SCL6A11, GABRG 3, and GABRB2) in its development [36].

## 4 DISCUSSION

In almost all these papers, ML algorithms showed promising results when combined with either pharmacogenomic information alone or clinical features.

The random forest algorithm seems to be the most adopted technique [26, 28-30, 32, 35]. The random forest classifier is a data mining method that offers superior classification performance than other innovative algorithms [47]. These properties have made random forests increasingly popular in the last few years, especially in psychiatry [48]. The expression “random forest” derives from being made of many trees; more specifically, random forest is a classifier consisting of a collection of tree-structured classifiers made of independent, identically distributed random vectors. Each tree casts a unit vote for the most popular class [49].

The use of clinical applications based on ML techniques considering pharmacogenomic data is not yet about to be used in everyday clinical use. All the studies adopted supervised ML technologies, where the outcome is already known, and artificial intelligence is trained to understand and predict such outcomes. Only Athreya and colleagues used unsupervised ML [30], but to test whether the distribution of symptom severity scores was expected; in a second step, they applied the trained random forest. Then, although ML currently has an investigative role, once it is trained on large numbers and a homogenous population, its diagnostic and predictive function will become more reliable and could be better used in clinical practice as a diagnostic tool [10-12].

In 2013 the FDA listed that pharmacogenomic testing should be used in early-phase clinical trials for the identification of suitable populations, cohorts, and individuals “that should receive lower or higher doses of a drug, or longer titration intervals, based on genetic effects on drug exposure, dose-response, early effectiveness and common adverse reactions” [50]. However, this innovative approach has not been widely adopted by pharmaceutical companies yet, both for the risk of reducing its potential market size and the lack of available extensive genomic data resources, data heterogeneity, and the absence of universal benchmarks [51].

Genomics represents a critical but small part of data needed for patient stratification, which involves heterogeneous biomedical, demographic, and sociometric data and effective predictive ML models. Despite not being designed for research application, substantial amounts of data within electronic health records have been proven for use through several notable studies in GWAS and phenome-wide association studies analysis [52]. Furthermore, studies on EHR-linked (Electronic Health Records) DNA biorepositories have successfully shown that integrating such pharmacogenomic and sociometric data can be helpful in predictive modeling for optimizing dosage and reducing dosing error [52]. By using clinically available information, such as age, gender, and education, healthcare providers and clinical researchers can identify better treatment options and patient responses to maximize efficacy and cost-effectiveness [52, 53].

However, several challenges are associated with the effective integration of EHR data with pharmacogenomics applications. For example, because of the high dimensionality of the EHR data structure, background noise, heterogeneity, shortage, incompleteness, random error, and systematic biases [54], extraction of relevant clinical phenotypes may require advanced computational models. Ongoing research in this field and recent advance in deep learning prove the potential of deep learning to overcome these difficulties and learn patient data representations that are useful for treatment response, adverse effects, and outcome prediction [51]. Recent applications include the extraction of general-purpose representations of patients from EHRs, often performed with generative models trained either on permanent or temporal data [51]. These models can uncover patterns in sparse, complex, heterogeneous datasets and produce surrogate patient phenotypes. Both are unsupervised, such as Deep Patient [54] and semi-supervised, for example, Denoising Autoencoder for Phenotype Stratification [55]. These models rely on an autoencoder network structure to model EHR data for deriving patient representations predictive of final diagnosis, and different outcomes (for example, drug response, mortality, adverse events, and hospitalization risk). As generative deep model development progresses quickly, applications of novel architectures, such as Generative Adversarial Networks, to EHR data are starting to emerge, demonstrating improved performance for the disease prediction [56] and risk prediction given treatment [57].

Even though ML seems promising, the application of deep learning algorithms in mental health is still in its first stages, with limited exploration. ML is yet treated as a black box by researchers, making this approach hard to be understood in terms of how and why these deep learning techniques work [58]. In this specific context, it is impossible to identify and differentiate which mechanisms are crucial in predicting therapy response and side effects depending on the diagnosis.

Moreover, most of the research on ML is still in the proof-of-concept stage, and there needs to be more real-life testing. Psychiatric diseases are a complex phenomenon with different aspects and variables (*i.e*., biological, social, psychological) concurring in causing such illnesses. Dang and colleagues showed that there is no standard way to gather high-quality data. There is difficulty in achieving the labels, which causes ML approaches to be uncertain, with the need for acknowledging the best practices in handling ML models [59]. Such difficulties and reasons might affect the application of ML models in everyday clinical practice. In order to make this tool effective and powerful, collecting a significant volume of high-quality data is essential. However, collecting a more detailed and high volume of data requires collaboration with institutions and a great effort, far from being easy [60].

The picture provided by our review is comprehensive yet must be considered, given its limitations. First, 8050 of 9180 enrolled subjects were affected by MDD, 684 had a psychotic disorder, 60 were diagnosed with BD, and only 38 showed autistic features. These results mean that the application of ML on pharmacogenomic, so far, has been tested mainly on a few mental disorders. Further studies must be conducted to explore the feasibility of this tool in tailoring pharmacological treatments for other diseases.

## 5 LIMITS IN THE CURRENT APPLICABILITY OF MACHINE LEARNING ALGORITHMS IN PSYCHIATRY

ML provides exciting potential for detecting, preventing, and treating psychiatric disorders. However, many factors limit its current use in research and practice [61-63]. Data is arguably the most apparent constraint in developing ML models for diagnosing and treating psychiatric disorders [61]. In psychiatry, we do not have the comfort of rich numerical datasets such as those available in intensive care units. Large datasets with diverse participants are needed to create accurate ML models. Best practice guidelines have been published for developing and reporting ML models in biomedical research [61].

Ethical concerns must be addressed before ML models are used in psychiatric disorders. Privacy and digital data security may affect a person's mental health. Furthermore, many concerns are related to the bias created in ML models that may disadvantage underrepresented groups [62]. Like humans, all ML models have some degree of error, and that error could be associated with significant clinical issues. Identifying Mental Health disorder risks based on digital data, such as that on social media, and providing help-seeking information may also be distressing for those who were unaware of their vulnerability [61]. The models are reliant on the availability of the specific predictors used to create them. The more intricate, timely, and costly the predictors are to collect and input into the ML algorithms, the harder they will be to utilize in practice [63]. ML models that rely on social media data may only be helpful for active users of those platforms [61]. Once the specific predictors have been collected, they need input into the ML algorithms. Ideally, this process would be automated, but it may be challenging if the ML models rely on predictors from different sources [64]. Simple interfaces should be created to allow humans to enter data without requiring extensive technical training [64].

Finally, and probably the most challenging issue to be technically addressed, is the clinicians' trust in the algorithm's capabilities [65]. Clinicians may not value the recommendations made by ML models or rely solely on them at the expense of their clinical judgment. An adequate balance between the algorithm's diagnostic power and the clinician's judgment is necessary for a field such as psychiatry. In mental health disorders, the diagnostical and therapeutical outcomes cannot always be mathematically defined, and the clinicians' human qualities are still often needed to reach a satisfactory clinical outcome [65].

## CONCLUSION

Precision psychiatry could already be considered a valuable clinical instrument in treating drug-resistant forms of many psychiatric disorders, such as MDD, BD, and psychoses. Indeed, it provides personalized therapy with improved efficacy and reduced adverse drug reactions by correlating genotype with clinical phenotype. Pharmacokinetic pharmacogenetic tests that combine different genomic variants show the most clinical utility. These tools are supposed to supplement rather than replace prescriber decisions, with clinical judgment remaining critical in decision-making. Pharmacogenomics could improve shared decision-making and risk-benefit analyses in medication selection. Moreover, the contamination of pharmacogenomics and ML in psychiatry may enlighten the development of clinical applications aimed at improving the choice of a drug treatment that could have an optimal outcome and tolerability. By the way, to reach the full potential of precision psychiatry, further research is needed to combine biodemographic data with multi-omics biomarkers and the whole spectrum of gene interactions using the latest AI computational strategies.

## Figures and Tables

**Table 1:** Studies’ characteristics.

**Authors**	**Type of Drug**	**Sample**	**Age, Years (SD)**	**Assessment Test**	**Design**
Eugene R.A. *et al.* 2018	Lithium	60 (20 ♂; 40 ♀) BD patients	♂; 41(10.8); ♀ 39 (13.1)	CGI-BP-S	Cohort study
Athreya P.A. *et al.* 2019	Citalopram/Escitalopram	1030 MDD patients	/	HDRS, QIDS-C	Cohort study (8 weeks)
Bi Y. *et al.* 2021	SSRI/SNRI/TCA/NaSSAs	610 MDD patients	35.96 (13.85)	HDRS, HAM-A	Cohort study (6 weeks)
Lin E. *et al.* 2020	Antidepressants (SSRI)	4223 MDD patients	43.7 (14.6)	HDRS	Cohort study (8 weeks)
Boloc D. *et al.* 2018	Antipsychotic (Amisulpride, Paliperidone, Risperidone, Risperidone LAI, Ziprasidone)	357 treated with antipsychotics	29.3 (10.0)	SAS	Cohort study (6months)
Fabbri C. *et al.* 2018	Antidepressants	671 patients (MDD)	/	MADRS, HDRS	Cohort study
Lee B.S. *et al.* 2018	Perphenazine, Olanzapine, Quetiapine, Risperidone, and Ziprasidone.	51 from the Clinical Antipsychotic Trials of Intervention Effectiveness (CATIE)	/	PANSS	Cohort study
Son W. *et al.* 2013	Typical antipsychotics	276 SCZ patients	46.29 (9.72) in TD SCZ *vs*. 43.47 (9.17) in nonTD SCZ	AIMS, RDC-TD	Case-control study
Kautzky A. *et al.* 2014	Antidepressants	225 MDD patients	50.99	HAM-D, MINI interview	Case-control study
Watanabe T. *et al.*	oxytocin	38 high functioning ASD	>20 years	ADOS, WAIS-R, ADI-R	Association study, data are taken from a clinical trial (with placebo)
Maciukiewicza M. *et al.* 2018	Duloxetine	186 MDD patients	46.7 (12.4)	MADRS	Association study, data are taken from a clinical trial (with placebo)
Borro M. *et al.*, 2021	Poly-therapy	200 MDD or BD patients with a depressive episode	56.9 (12)	/	Cohort study
Joyce J.B. *et al.*, 2021	Citaloprma, escitalopram, bupropion, venlafaxine, mirtazapine, placebo	375 MDD patients	42 (12.4)	QIDS-C	Association study, data are taken from a clinical trial (with placebo)
Taliaz D. *et al.*, 2021	Citalopram, sertraline and venlafaxine	530 MDD patients	/	QIDS-C and HDRS	Association study, data are taken from a clinical trial (with placebo)

**Abbreviations**: Bipolar Disorder (BD); Schizophrenia (SCZ); Clinical Global Impression Scale for Bipolar Disorder-Severity (CGI-BP-S); Hamilton Depression Rating Scale (HDRS); Quick Inventory of Depressive Symptomatology (QIDS-C); Hamilton Depression Rating Scale (HDRS); Hamilton Anxiety Rating Scale (HAM-A); Simpson-Angus Scale (SAS); Montgomery–Åsberg Depression Rating Scale (MADRS); Positive and Negative Syndrome Scale (PANSS); Abnormal Involuntary Movement Scale (AIMS); Research Diagnostic Criteria for TD (RDC-TD); Autism Diagnostic Observation (ADOS); Wechsler Adult Intelligence Scale-Revised (WAIS-R); Autism Diagnostic Interview-Revised (ADI-R); Major Depressive Disorder (MDD); Autism Spectrum Disorder (ASD); Standard Deviation (SD); Selective Serotonin Reuptake Inhibitor (SSRI); Serotonin Noradrenalin Reuptake Inhibitor (SNRI); Tricyclic antidepressant (TCA); Noradrenergic and Specific Serotonergic Antidepressants (NaSSAs).

**Table 2:** Algorithms’ and genomics’ features.

**Authors**	**Machine Learning Algorithm**	**Tools**	**Main Hypothesis**	**Outcome**	**Genes**
Eugene R.A. *et al.* 2018	Decision Tree Random Forest Machine Learning techniques.	DNA microarray from Lithium Treatment-Moderate dose Use Study placed in the National Center for Biotechnology Information (NCBI) Gene Expression Omnibus (GEO); Illumina HumanHT12 V4.0 expression, Beadchip GPL10558 platform file to associate gene names and descriptions.	Transcriptome-level gene signatures are differentially expressed between male and female bipolar patients, before lithium treatment.	Pre-treatment gender- and gene-expression-based predictive model selective for classifying male lithium responders with a sensitivity of 96% using 2-genes and female lithium responders with sensitivity = 92% using 3-genes.	RBPMS2 and LILRA5 genes classify male lithium responders with AUROC: 0.92 and the ABRACL, FHL3, and NBPF14 genes.
Athreya P.A. *et al.* 2019	Unsupervised machine learning to test whether the distribution of symptom severity scores was normal. Random forests based on baseline depression severity and pharmacogenomics data to predict SSRI response and remission.	A genome-wide association study for PGRN-AMPS plasma metabolites associated with SSRI response (serotonin) and baseline MDD severity (kynurenine) identified single nucleotide polymorphisms (SNPs)	Machine learning-based algorithms may predict selective serotonin reuptake inhibitors in MDD patients	Supervised machine-learning methods trained using SNPs and total baseline depression scores predicted remission and response at 8 weeks with the area under the receiver operating curve (AUC) > 0.7 (*P* < 0.04) in PGRN-AMPS patients, with comparable prediction accuracies > 69% (P ≤ 0.07) in STAR*D and ISPC	Baseline kynurenine in MDD and DEFB1, ERICH3, AHR, and TSPAN5 were tested as predictors. DEFB1_2 (rs2741130) and DEFB1_1 (rs5743467) SNPs—biomarkers identified. The top SNPs for the response for men were the TSPAN5 SNPs, followed by the DEFB1_1 and DEFB1_2 SNPs. DEFB1_1 SNP, followed by DEFB1_2 SNP
Bi Y. *et al.* 2021	Random forest is employed to screen factors that predict antidepressant efficacy from multidimensional variables.	All SNPs were genotyped by mass spectrometers using MassArray Analyzer 4 system. All probes and primers were predesigned by the MassARRAY Assay Design 3.0 software. The sample DNA was amplified by a multiplex PCR reaction. The extension products were analyzed by Matrix-Assisted Laser	Genetics, cognitive, neuroendocrine, as well as personality factors, are all intrinsically linked and contribute to the diversity of treatment outcomes.	SSRI and SNRI are better treatments than TCA and NaSSA in the Chinese population. Citalopram and venlafaxine were more effective than mirtazapine. rs929377-rs6191-rs32897 located in the HPA pathway was significantly associated with the treatment outcome of fluoxetine.	rs6354 and rs12150214 in gene SLC6A4; rs3847621 in gene SLC1A2; rs478962 in gene GRIA1; rs9870680 in gene GRM7; rs6323 in gene MAOA; rs1137070 in gene MAOA
Lin E. *et al.* 2020	A wrapper-based feature selection algorithm was adopted, where the feature selection algorithm acts as a wrapper around the predictive algorithm. It was integrated with a boosting ensemble model.	For all subjects, they performed single nucleotide polymorphism SNP genotyping by using Illumina HumanOmniExpressExome BeadChips in the International SSRI Pharmacogenomics Consortium	Chose the SSRI response relying on 10 genetic variants and 6 clinical variables.	The average value of the receiver operating curve (AUC) for the boosting ensemble prediction model with the wrapper-based feature selection algorithm was 0.8122 (standard deviation = 0.0702) by using the selected 15 features. For forecasting antidepressant remission, the average value of AUC was 0.8111 (standard deviation = 0.0691) by using the original 16 biomarkers.	ARNTL rs11022778, CAMK1D rs2724812, GABRB3 rs12904459, GRM8 rs35864549, NAALADL2 rs9878985, NCALD rs483986, PLA2G4A rs12046378, PROK2 rs73103153, RBFOX1 rs17134927, and ZNF536 rs77554113 SNPs
Boloc D. *et al.* 2018	Supervision methods of class prediction based on machine learning (ML) were applied. ML is trained using the Discovery Sample of the SNPassoc R package. Support vector machine, Naive Bayes and Random Forest were used.	Real-time PCR using TaqMan allelic discrimination predesigned assays.	Genetic factors implied in Extrapyramidal symptoms during antipsychotic therapy may be detected with ML techniques.	The three machine learning methods showed a better EPS prediction. The Naive Bayes achieved the best result.	AKT1 (rs1130214, rs74090038, rs33925946); FCHSD1 (rs1421896, rs34798770); DDIT4 (rs1053639, rs4747241, rs474742, rs10823911); Raptor (rs34726568, rs9899898, rs9915667)
Fabbri C. *et al.* 2018	Five machine learning models (neural networks, recursive partitioning, learning vector quantization, gradient boosted machine and random forests) were adopted.	/	FKBP5 and CACNA1C polymorphisms may play a role in antidepressant response and TRD (treatment-resistant depression).	In all original samples response and remission at week 4 or 6 were investigated according to standard definitions (response was defined as a decrease of at least 50% in the HDRS-21 or the MADRS, while remission was defined as HDRS ≤ 7 or MADRS < 10).	ANK3 (rs1049862); CACNA1C (rs2283326, rs10848635, rs11062157); FKB5 (rs9470080, rs9368882, rs38000373, rs1360780) ; CACNA1C (rs2283326, rs1006737, rs10848635); CACNB2 (rs2799573); FKBP5 (rs3800373, rs1360780)
Lee B.S. *et al.* 2018	A classical clustering algorithm called the partitioning around medoids (PAM) algorithm and, for the distance measure, the dissimilarity measure of Gower.	/	A computational algorithm may predict treatment response with antipsychotic medication among patients with schizophrenia.	The model provided a promising prediction for Ziprasidone by 13 SNPs and 53 baseline variables.	rs10803138, rs11682175, rs6704641, rs6704768, rs215411, rs1106568, rs12522290, rs4129585, rs2514218, rs2239063, rs4702, rs12325245, and rs9636107, rs4846033, rs10911902, rs9309325, rs1569351, rs4568102, rs1380272, rs1495716, rs9295938, rs9400690, rs16917897, rs297257, rs9512730, rs942348, rs17070578, rs17095545, rs7144633, rs16977195, rs234993, rs151222, rs17455133, rs2824301, rs10521865, rs2159767, rs2536589, rs952515.
Son W. *et al.* 2013	Multifactor dimensionality reduction (MDR) Stage 1 involves choosing the best combination of multifactor, and MDR stage 2 involves classifying the combinations of genotypes into high-risk and low-risk groups.	Genomic DNA was isolated using NucleoSpin^®^ Blood DNA Extraction Kit (Macherey-Nagel, Germany) according to manual procedures.	ML techniques may detect which polymorphisms of the GABA transporter gene are associated with tardive dyskinesia (TD).	SCL6A11 genotypes distribution showed a significant difference between the TD and non-TD patients (P 0.049). These analyses provided significant evidence for gene-gene interactions (SCL6A11, GABRG 3 and GABRB2) in the development of TD.	Three polymorphisms in SLC6A11 (rs4684742), GABRG3 (rs2061051) and GABRB2 (rs918528)
Kautzky A. *et al.* 2014	A random forest algorithm was adopted.	The SequenomiPLEX assay of Cogenics was used to obtain genotypes, deploying locus-specific PCR primers as well as allele-specific detection primers according to the protocol of the MassARRAYAssayDesign software.	ML algorithm might be adopted to spotlight SNPs and clinical variables related to treatment response.	About 62%of patients exhibiting the allelic combination of GG-GG-TT for rs6265, rs7430 and rs6313 of the BDNF, PPP3CC and HTR2A genes, respectively, and without melancholia showed a HAM-D decline under 17 compared to about 34% of the whole study sample.	Polymorphisms from the BDNF gene (rs6265, rs11030101and rs11030104) were chosen. SNPs from the PPP3CC gene (rs7430, rs10108011). Two SNPs from the ST8SIA2 gene (rs3784732, rs8035760). Two SNPs from the COMT gene (rs174696, rs4680). Two SNPs from the HTR2A gene.
Watanabe T. *et al.* 2017	Support vector regression (SVR) machine learning.	Among 27 OXTR SNPs of interest were selected 21 based on Affymetrix Genome-Wide Human SNP Array 6.0.	Among 27 OXTR SNPs of interest were selected 21 based on Affymetrix Genome-Wide Human SNP Array 6.0.	Major alleles of several prominent OXTR SNPs-including rs53576 and rs2254298-were found to have dissociable effects on the oxytocin efficacies.	The best selection was among six types of efficacy in 21 alleles for OXTR.
Maciukiewicz M. *et al.* 2018	Two machine-learning algorithms were utilized: classification-regression trees (CRT) and linear support vector machine (SVM)	Infinium PsychArray BeadChip by Illumina (“PsychChip”)	The current investigation aims to use supervised machine learning (ML) to build predictive models of duloxetine outcome in a small MDD cohort with a standard depressive assessment with available genome-wide data.	none of the pairs performed significantly better than chance (accuracy *p* > .1). The best performing SVM fold was characterized by an accuracy = 0.66 (*p* = .071), sensitivity = 0.70 and a sensitivity = 0.61.	rs2036270 RARB intronic; rs7037011 19 kb 3′ of RP11-29B9.1; exm775913 (rs1138545) TNC missense;; rs1107372 2.8 kb 3′ of TNC; rs11136977 RP11-124B13.1; rs11581838 LINC00466 intronic; rs11843926 5′ of SLC10A2; rs1347866 61 kb 5′ of; AC062021.1; rs16932062 6.8 kb 3′ of TNC; rs1999223 7.5 kb 3′ of TNC; rs2710664 VSNL1 intronic; rs39185 THSD7A; rs4520243 FCN2 synonymous; rs4685865 5′ of ARL8B; rs4777522 3′ of MIR630; rs4954764 54 kb 5′ of AC062021.1; rs60230255 3 VSNL1 intronic; rs6550948 5′ of RARB; rs972016 5′ of RARB
Borro M. *et al.* 2021	Drug-PIN programme based on multi-pass analysis algorithm.	/	Drug-PIN might help clinicians to optimise pharmacological therapies in patients diagnosed with major depressive disorder or depressive episodes in bipolar disorder with treatment failure in at least three psychopharmacological therapies.	Drug-PIN replicates the output of a counselling process, allowing for optimisations of the assessment of the risk and efficacies of polytherapy.	ABCB1 (rs1128503, rs1045642); ABCC1 (rs45511401); ABCC2 (rs8187710, rs17222723, rs717620); ABCG2 (rs2231142); SLCO1B1 (rs4363657, rs4149056); SLC15A2 (rs2257212); 5-HTT; 5HTT-LPR; CYP1A1 (rs1048943); CYP1A2 (rs2069514, rs762551); CYP2A6 (rs28399433, rs1801272); CYP2B6 (rs2279343, rs3745274, rs3211371, rs28399499); CYP2C8 (rs11572103, rs1058930); CYP2C9 (rs1799853, rs1057910); CYP2C19 (rs6413438, rs12248560, rs4244285, rs4986893, rs28399504, rs56337013, rs72558186); CYP2D6 (rs1065852, rs28371706, rs16947, rs61736512, rs1080985, rs35742686, rs3892097, rs28371725, rs5030655, rs5030867, rs5030656, rs72549351, rs72549354); CYP3A4 (rs2740574, rs35599367); CYP3A5 (rs776746); COMT (rs4680, rs4633, rs4818); EPHX1 (rs2234922, rs1051740); NAT1 (rs5030839, rs56172717, rs56379106, rs4986782); NAT2 (rs1801280, rs1799930, rs1799931); TPMT (rs1800462, rs1800460, rs1142345); UGT1A1 (rs8175347); UGT2B17 (Gene deletion); DRD2 (rs1800497, rs1799732, rs1801028); DRD3 (rs6280); HTR2A (rs6314, rs7997012, rs6311); HTR2C (rs6318); OPRM1 (rs1799971)
Joyce J.B. *et al.*, 2021	Linear and non-linear algorithm; tree-based algorithm.	Liquid chromatography electrochemical coulometric array (LC-ECA) metabolomics platform.	Augmenting clinical measures (*e.g*., symptom severity scores) with multiple biological measures (*e.g*., metabolomics and genomics) might improve the predictability of response to combined antidepressant therapies.	Integrating specific metabolites and SNPs achieves accurate predictions of treatment response across classes of antidepressants.	TSPAN5(rs10516436) ERICH3(rs696692), DEFB1(rs5743467, rs2741130 and rs2702877) and AHR(rs17137566).
Taliaz D. *et al.*, 2021	Support vector machine (SVM) with a linear kernel, eXtreme Gradient Boosting (XGBoost), Random Forest, and Adaptive Boosting (AdaBoost).	/	An application based on integrated multimodal data might enable a more comprehensive and accurate prediction for the treatment of depression and will pave the way for similar analyses of accumulating data by new technologies.	Applying ML to datasets with genetic, clinical, and demographic features to improve accuracy in antidepressant prescription	8120 SNPs

**Abbreviations**: Machine Learning (ML), single-nucleotide polymorphism (SNP).

## LIST OF ABBREVIATIONS

ASD: Autism Spectrum Disorder
BD: Bipolar Disorder
MDD: Major Depressive Disorder
ML: Machine Learning
RCT: Randomized Control Studies
TD: Tardive Dyskinesia
